# THE ROLE OF RELATIONSHIP FACTORS IN HARMFUL CAREGIVER BEHAVIORS: OLDER CHILDREN CARING FOR PARENTS WITH DEMENTIA

**DOI:** 10.1093/geroni/igac059.2291

**Published:** 2022-12-20

**Authors:** Elizabeth Gallagher, Kathrin Boerner, Yijung Kim, Kyungmin Kim, Daniela Jopp

**Affiliations:** University of Massachusetts Boston, Boston, Massachusetts, United States; University of Massachusetts Boston, Boston, Massachusetts, United States; The University of Texas at Austin, Austin, Texas, United States; Seoul National University, Seoul, Seoul-t'ukpyolsi, Republic of Korea; University of Lausanne, Lausanne, Vaud, Switzerland

## Abstract

Elder abuse by family caregivers is an often-overlooked phenomenon that affects many older adults. Especially, retirement-aged children caring for their oldest-old parents with dementia may be at greater risk of engaging in harmful or abusive behaviors, given their own age-related health issues and other competing caregiving demands. Most of the elder abuse literature has focused on general demographic predictors of elder abuse, regarding the caregiver, care recipient, and the care environment. Less attention has been paid towards relationship factors, which may play a large role among these parent-child dyads. This study examined how relationship factors are associated with potentially harmful caregiver behaviors (PHCB; e.g., screaming), which have been identified as “early warning signs” for elder abuse. Relationship factors of interest include positive and negative relationship quality measured by caregivers’ mean scores on the support and conflict subscales on the Quality of Relationship Inventory (QRI). We conducted in-depth interviews with 88 caregivers (65+) who are caring for their parents with dementia (90+) as the part of the Boston Aging Together Study. Regression models revealed that relationship conflict was significantly associated with higher levels of PHCB, accounting for caregiver, care recipient, and care environment characteristics. The creation of screeners to identify “high conflict” care dyads could prove useful in the early detection and intervention of potential elder abuse cases, given that caregivers may be more willing to report negative aspects of their relationship (e.g., fighting) than more obviously harmful or abusive behaviors.

